# Intestinal Microbes in Autoimmune and Inflammatory Disease

**DOI:** 10.3389/fimmu.2020.597966

**Published:** 2020-12-23

**Authors:** Wan-Jung H. Wu, Daniel F. Zegarra-Ruiz, Gretchen E. Diehl

**Affiliations:** ^1^ Immunology Graduate Program, Baylor College of Medicine, Houston, TX, United States; ^2^ Immunology Program of the Sloan Kettering Institute, Memorial Sloan Kettering Cancer Center, New York, NY, United States

**Keywords:** multiple sclerosis, autoimmunity, inflammation, inflammatory bowel disease, type 1 diabetes, rheumathoid arthritis, systemic lupus erythematosus, intestinal microbiota

## Abstract

Autoimmune diseases and chronic inflammatory disorders are characterized by dysregulated immune responses resulting in excessive and uncontrolled tissue inflammation. Multiple factors including genetic variation, environmental stimuli, and infection are all thought to contribute to continued inflammation and pathology. Current evidence supports the microbiota as one such factor with emerging data linking commensal organisms to the onset and progression of disease. In this review, we will discuss links between the microbiota and specific diseases as well as highlight common pathways that link intestinal microbes with multiple autoimmune and inflammatory diseases.

## Introduction

Increases in autoimmune and inflammatory diseases are a major health problem currently affecting over 200 million people worldwide and represent a leading cause of death for women under 65 (1, 2). Better understanding of factors that affect disease progression and initiation will lead to new ways to address these important health issues.

In the human body, the microbiota dynamically interacts with the host at all barrier sites with the largest load of microbes residing within the intestine (3). Commensals coevolved with humans and provide multiple benefits including facilitating nutrition and xenobiotic metabolism, enhancing barrier function, inhibiting pathogens, and modulating immunity (3). Alteration in the microbiota composition is linked to dysregulated immunity and is associated with inflammatory and autoimmune diseases (4–9).

While individual studies find a number of disease-associated changes, how these changes relate to disease initiation or amplification are still being elucidated. Importantly, understanding host regulation by intestinal microbes or of microbial physiology have led to greater understanding of a number of diseases. For example, microbial factors such as metabolites can play an important role in modulating intestinal and systemic inflammation and a subset of metabolites are linked to multiple diseases (10). Short-chain fatty acids (SCFAs), which are converted from dietary fiber and as the main energy source for colonocytes, directly support intestinal epithelial health (11). SCFAs also promote differentiation of regulatory T cells (Tregs) supporting an anti-inflammatory environment within the gut and at distal sites (12–15). While many types of microbes can generate SCFA, the main producers are Firmicutes and Bacteroidetes and increased proportion of these organisms is associated with human health (16). Dietary factors such as fiber can also shape the microbial community by modifying the metabolic landscape resulting in microbial compositional changes that can modulate diseases (17, 18). Common associations with metabolites highlight how common metabolic pathways utilized by distinct microbes could modulate disease. They also give clues to common pathways that could be manipulated to treat these diseases.

Additionally, in many of these diseases, increased microbes or microbial products can be found in the blood indicating that changes to the intestinal barrier may be a common feature (19–21). However, whether these changes are causative or a consequence of disease development remains to be seen (22).

On the host side, several pathways associated with microbiota-regulated immune responses are linked to autoimmune and inflammatory diseases (23–25). Mutations in HLA-DR, toll like receptors (TLRs), inflammasome, and autophagy components are associated with multiple diseases where they lead to dysregulated immune responses and increased inflammation (26–29).

In this review, we will discuss association of the microbiota with pathways involved in the pathogenesis of inflammatory bowel disease (IBD), systemic lupus erythematosus (SLE), rheumatoid arthritis (RA), multiple sclerosis (MS), and type I diabetes (T1D) to highlight commonalities between diseases as well as point out disease specific associations.

## Inflammatory Bowel Disease

IBD is characterized by dysregulated immune responses against the microbiota leading to chronic inflammation in the gastrointestinal (GI) tract. The major forms of IBD are ulcerative colitis (UC), which is limited to the colon, and Crohn’s disease (CD), which can affect tissue throughout the GI tract (30). In IBD patients, there are reductions in potentially anti-inflammatory microbes such as Bacteroidetes, *Lachnospiraceae* (16), and *Faecalibacterium prausnitzii* (31, 32) alongside increases in potentially inflammatory microbes such as Proteobacteria and *Ruminococcus gnavus* (30, 33–39). Further, increased mucosa-associated bacteria (16, 40) results in greater contact between gut microbes and immune system and leads to anti-bacterial immunity associated with IBD pathogenesis (41–45).

In humans, over 240 genetic loci are associated with risk for IBD (23, 46–49). Gene mutations in pathways related to interactions with the microbiota highlight common mechanisms for disease development (23). Mutations are found in genes associated with microbial recognition including nucleotide-binding oligomerization domain-containing protein 2 (NOD2); anti-inflammatory mechanisms including IL-10 and IL-10 receptor (50–53) and barrier repair including IL-22 (54, 55). Many have found these pathways are induced by the microbiota and the microbiota is important for barrier repair in mouse models of disease (56–62). However, microbes also drive pathology and rederivation to germfree is protective in T cell dependent models (59, 63).

In addition, diet and dietary metabolites are critical factors in IBD pathogenesis (64). In IBD patients, specific bacteria, such as butyrate producers *Faecalibacterium prausnitzii and Roseburia hominis* are decreased (32, 65). The crucial roles of diet and dietary metabolites are shown in multiple mouse models where high fiber diets or direct administration of SCFA are beneficial while loss of the SCFA receptor, Gpr43, is pathogenic (12–15). Tryptophan metabolites can also mitigate colitis severity. These are ligands for the aryl hydrocarbon receptor (AhR), which activates IL-22 and IL-10 production and is negatively associated with colitis (66–69). A tryptophan-free diet exacerbates pathology in colitis models (70), whereas *Lactobacillus bulgaricus*, an AhR-activating bacterium, ameliorates pathology (66, 71). Secondary bile acids are additional metabolites with both pro- and anti-inflammatory functions that can promote differentiation of Tregs or Th17 cells within the intestine and in peripheral sites (72, 73). Bile acids can also regulate intestinal bacterial growth by enhancing biofilm formation thereby increasing colonization by pathogens such as vancomycin-resistant *Enterococcus* in mice (74). These studies together highlight the complex interaction between the host, diet, and intestinal microbes that can underlie alterations in disease pathology.

As evidence supports the potential for the microbiota in maintaining intestinal homeostasis and preventing inflammation, there is great interest in utilizing microbes as treatment for IBD patients. The administration of probiotics shows success in animal models (75, 76) and some patients (77, 78). However, broad scale benefits are yet to emerge (78). This may be due to the genetic complexity or other environmental factors associated with IBD. Another alternative is fecal microbiota transplants (FMT), which are utilized successfully to treat *C. difficile* infection (79). Several trials demonstrate success in some UC patients (80, 81). FMT increases microbiota diversity in responders and non-responders (80), demonstrating that increased diversity alone is not sufficient for benefit. Interestingly, recent work found expanded intestinal bacteriophages in patients who did not respond after FMT with bacteriophages exacerbating colitis in animal models (82). More work needs to be done to understand how FMT can shape the recipient’s microbial community to define if this method can broadly ameliorate diseases.

Together, work in IBD demonstrates myriad ways the microbiota interacts with the host to regulate local inflammation and suggests a number of microbiota-related pathways to target for treating this disease. Understanding affected pathways in IBD have also improved understanding of how microbes impact other inflammatory and autoimmune diseases and will lead to a broader understanding of how to utilize the microbiome to improve patient outcomes.

## Systemic Lupus Erythematosus

SLE patients suffer from production of autoantibodies and proinflammatory cytokines that cause disease in multiple organs including skin, blood, and kidneys with many environmental influences, including the gut microbiota (83). SLE patients exhibit intestinal and oral dysbiosis. As with other autoimmune diseases, studies find decreased bacterial diversity correlated with disease activity (6). Oral and gut microbiota from SLE patients are enriched in the family *Lactobacillaceae*, with *Bifidobacteria* and Clostridiales decreased in the intestine (84–86).

Further, antibodies and T cells from SLE patients recognize bacterial antigens from the oral, intestinal, and skin microbiota including *Propionibacterium propionicum* and *Bacteroides thetaiotaomicron* (6, 84, 85, 87). Molecular mimicry is a possible link between the microbiota and SLE. One of the most common autoantibodies associated with SLE targets the broadly expressed RNA binding protein Ro60 (88). Antibodies against Ro60 are commonly found before SLE symptoms develop (89). Some human commensals produce proteins similar to human Ro60 and, although these bacteria are found in both healthy donors and lupus patients, only lupus patients have antibodies and T cells reactive with human Ro60 and microbial Ro60 orthologs (87). In addition, in SLE patients, disease severity correlated with *R. gnavus* enrichment. Further, SLE patients with severe disease had IgG antibodies that recognized cell wall lipoglycans from a subset of *R. gnavus* strains. Importantly, auto-DNA antibodies from SLE patients with lupus nephritis were cross-reactive with *R. gnavus* lipoglycans (6).

Multiple spontaneous and inducible mouse lupus models have given great insight into how microbiota changes regulate pathology. Intercross of mouse strains NZW with BXSB results in spontaneous lupus-related antiphospholipid syndrome and liver damage, predominately in male mice, due to an extra copy of the TLR7 gene. In these mice, *Enterococcus gallinarum* translocates to the liver and triggers autoimmune responses. Depletion of this pathobiont with vancomycin suppressed bacterial translocation, autoreactive T cells, and autoantibodies. Monocolonization of germfree mice with *E. gallinarum* increased gut permeability, plasmacytoid dendritic cells (pDCs) and Th17 cells in the intestine lamina propria and mesenteric lymph nodes, exacerbating disease and mortality (90).

Bacterial metabolites also modulate SLE as seen with *Lactobacillus* which, as discussed above can modulate intestinal inflammation by producing AhR-activating ligands (66, 71). In mouse models, and in contrast with IBD, a high protein diet with a high tryptophan content is associated with increased pathology by promoting anti-double stranded DNA autoantibody production and increased T follicular helper (Tfh) cells (91). A metabolic screening from feces of lupus prone mice homozygous for the NZM2410 lupus susceptibility quantitative trait loci (Sle1, Sle2, and Sle3) showed increased intestinal tryptophan-derived bacterial metabolites with enriched fecal *Lactobacillus* (91).

In contrast, and similar to IBD, a high fiber diet is associated with improved outcomes in mouse lupus models (86). In a TLR7-dependent model, there was outgrowth of *Lactobacillus reuteri*, which then translocated to the mesenteric lymph node, spleen, and liver. Translocation led to increased pDC production of type I interferon (IFN-I), exacerbating disease pathogenesis and mortality. Treatment with SCFAs or a high fiber diet suppressed *L. reuteri* outgrowth and translocation, reducing excess IFN-I and ameliorating disease (86). This example shows both direct and indirect effects of gut commensals on disease progression.

Together, these results demonstrate that the gut microbiota can modulate lupus pathogenesis by molecular mimicry, changes in bacterial translocation, metabolites, or microbe-microbe competition. Each can result in a dysregulated immune response in distal tissues including Th17 cell and pDC recruitment and activation of IFN-I pathways that together amplify disease.

## Rheumatoid Arthritis

RA is a chronic synovial inflammation characterized by immune infiltration in the joints due to lost tolerance including B and T cell responses against self-proteins with a citrulline residue leading to cartilage degradation and bone erosion (92). In a subset of RA patients, bacterial DNA and peptidoglycan–polysaccharide complexes are found in the synovium (93). RA patients exhibit oral dysbiosis, characterized by enrichment of *Porphyromonas gingivalis* and *Lactobacillus salivarius* and intestinal dysbiosis with increased Gram-positive bacteria (94, 95). These changes in the oral and gut microbiota are linked to clinical variations in RA (93). Increased abundance of *Lactobacillus* correlated with increased total IgG titers, while other oral microbes such as *Prevotella* spp. correlated with rheumatoid factor (95). *Prevotella copri* is enriched in fecal samples of patients and individuals at risk for RA. A subset of RA patients has *P. copri*-specific Th1 and Th17 cells along with IgG and IgA antibodies which correlates with increased proinflammatory cytokine levels and more severe disease (95–97). Interestingly, RA therapies partially restores the microbiota to more closely resemble one found in healthy controls (95).

In mice, TLR2 and TLR4 engagement modulates autoimmune arthritis (98). IL-1 receptor antagonist–knockout (*Il1rn^-/-^*) mice spontaneously develop autoimmune arthritis due to uncontrolled IL-1 signaling (99). Disease progression is delayed in germfree *Il1rn^-/-^* mice (98). A single injection of a TLR2 agonist or monocolonization with *Lactobacillus bifidus* was sufficient to restore pathogenesis (98). However, as with other diseases, there are complex interactions between these pathways. *Il1rn^-/-^* mice lacking TLR2 exhibited exacerbated disease with increased bone destruction mediated by Th1 cells, suggesting a dual role for TLR2 in disease (98). BALB/c ZAP-70(W163C)-mutant (SKG) mice spontaneously develop chronic arthritis due to a naturally occurring mutation of the ZAP-70 gene, a signal transduction molecule downstream of the T cell receptor (100, 101). Germfree SKG mice do not develop disease (100). Conventionalization with altered Schaedler flora (ASF), a defined community of eight bacteria including *Lactobacillus* species, was sufficient to induce arthritis, supporting the role of gut microbes in pathogenesis (100). Further supporting microbiota shifts found in RA as amplifying disease, conventionalization of SKG germfree mice with fecal samples from RA patients elicited more severe arthritis with higher levels of IL-17A as compared to fecal samples from healthy controls (97). Similarly, *P. copri*-monocolonized SKG mice have exacerbated disease with increased Th17 cells (97). Colonization with Segmented filamentous bacteria (SFB), a Th17 cell inducing mouse commensal, exacerbates a K/BxN autoimmune arthritis model (in which KRN T cells recognize glucose-6-phosphate isomerase) by expanding Tfh cells, which promote the production of autoantibodies involved in RA (102). These data show that gut microbes can modulate immune responses involved in RA such as Th1 and Th17 cells recruitment and expansion exacerbating the inflamed tissue environment.

## Multiple Sclerosis

MS patients suffer from autoimmune responses against the brain and spinal cord due to T cell targeting of oligodendrocytes resulting in demyelination and axonal loss (103). MS patients exhibit intestinal dysbiosis with increases in the Euryarchaeota and Verrucomicrobia phyla. Specifically, *Methanobrevibacter smithii* and *Akkermansia muciniphila* are enriched in the stool of patients and their abundance decreased after treatment (8). In addition, reduced levels are found of bacteria belonging to the Clostridia clusters XIVa and IV and Bacteroidetes, microbes well known to produce SCFA and induce Treg cells (104, 105).

As with other diseases, in the mouse model of MS, experimental autoimmune encephalitis (EAE), pathology is ameliorated in germfree mice with lower levels of IFN-γ and IL-17A and increased Treg cells (106). Interestingly, in the relapsing remitting MS mouse model, in which CD4^+^ T cells are specific for myelin oligodendrocyte glycoprotein (MOG), transfer of intestinal microbes from MS patients but not from healthy monozygotic twins increased incidence of disease due to decreased T cell IL-10 production (107). *A. muciniphila* also affects T cell differentiation by inducing Th1 differentiation in PBMCs from both healthy donors and MS patients, potentially contributing to the proinflammatory environment in MS (108).

Similar to IBD and in contrast with SLE, in mouse models tryptophan can protect from pathogenesis. Colonization of mice in the EAE model with *Lactobacillus reuteri* through its conversion of tryptophan into AhR agonists, activates IFN-I responses in astrocytes and limits disease severity (109). Another *Lactobacillius* species, *L. murinus* reduces EAE severity by inhibiting Th17 cell differentiation (110). Intestinal colonization by *L. murinus* is suppressed by a high salt diet, which also amplifies disease (110). Together these studies demonstrate a gut/brain axis in which gut microbes and metabolites modulate immune responses including innate and adaptive immunity at distal sites to influence disease onset and severity.

## Type 1 Diabetes

Immune destruction of pancreatic β-cells by islet-specific autoreactive CD8^+^ T cells results in lost insulin production and T1D (111). A longitudinal human study analyzing stool samples from the Environmental Determinants of Diabetes in the Young (TEDDY) cohort identified reduced microbial pathways related to fermentation and synthesis of SCFAs as well as decreased microbial diversity as well as reduced *Bifidobacteria* and *Lachnospiraceae* and overabundance of *Blautia*, *Rikenellaceae*, and *Ruminococcus* in patients who progressed to T1D (9, 112). In a similar cohort, children who progress to T1D show changes in the Bacteroidetes/Firmicutes ratio and increased *Bacteroides ovatus* (9, 112–115).

In contrast with other disease models, germfree non-obese diabetic (NOD) mice have increased islet destruction demonstrating that in diabetes, microbes may limit disease severity (116). However, some microbes are likely pathogenic as depletion of Gram-negative gut microbes in neonatal mice results in decreased diabetes incidence with fewer IFN-γ producing T cells (117). Supporting the complicated pro- and anti-inflammatory signals downstream of microbes, in contrast with IBD, loss of MyD88 protects specific pathogen free (SPF) or ASF colonized NOD mice from diabetes however rederivation to germfree, restores disease incidence (116). As in SLE, bacterial translocation can be a factor in T1D pathogenesis. In a model of streptozotocin (STZ)-induced T1D, gut microbial translocation to the pancreatic lymph node led to recognition of bacterial MDP by the intracellular NOD2 receptor resulting in increased number of Th1 and Th17 cells and increased islet destruction (118). Similar to the enrichment seen in children that develop T1D, STZ-treated mice also had increased intestinal *Bacteroides* (114, 118). Gut microbiota also plays a role in sex differences in autoimmune diseases. In SPF NOD mice, female mice have a higher incidence of disease than male mice with no differences between the sexes in germfree mice (119). Cecal microbiome transplants from male to female mice reduced islet inflammation and autoantibody levels due to microbiome changes along with hormonal and metabolic changes downstream of elevated testosterone (119).

In NOD mice, as with IBD and MS, SCFAs, notably butyrate, decreased the incidence and severity of diabetes with reduced frequency of autoimmune CD8^+^ T cells and B cells and increased Tregs and IL-10 production (120). Treatment with SCFAs increased the abundance of *Bacteroides*, which protected against disease when transplanted to germfree NOD mice (120).

Together, in mouse models, gut microbes and gut microbial metabolites can modulate immune responses involved in T1D including pancreas T cell infiltration as well as shaping the balance between pro- and anti-inflammatory T cell responses, thereby influencing disease onset and severity.

## Conclusion

In this review, we provided examples of mechanistic ways microbes can alter disease pathology in IBD, SLE, RA, MS, and T1D models with microbes playing a role in pathology of additional autoimmune diseases (121–124). While we focused on bacteria, emerging data suggests potential roles for yeast and enteric viruses in modulating immune responses and autoimmune and inflammatory disease (125–128).

We have highlighted disease specific interactions as well as numerous common links between the microbiota and human disease (**Table 1**). Common associations relate to microbial behaviors such as translocation or microbial metabolites that are shared between multiple microbes. Understanding these common functions and as the host pathways regulated by the microbiota will enable for identification of targetable pathways to treat multiple autoimmune and inflammatory disease.

**Table 1:** Summary table for the relationship between bacteria and autoinflammatory and autoimmune diseases.

Bacteria - Family	Bacteria - Species	Disease	Abundance	Human Subjects	Ref.	Mouse Model	Ref.	Effect	Mechanism/Pathway (Metabolite)
***Akkermansiaceae***	***A. muciniphila***	MS	Enriched	60P and 43HC	(8)				
***Bacteroidaceae***	***B. ovatus***	T1D	Enriched	8P and 24HC	(114)				
	***B. thetaiotaomicron***	SLE	Enriched			TLR7 overexpression	(87)	Exacerbates	Molecular mimicry/Autoantibodies
***Bifidobacteriaceae***	***Bifidobacteria***	SLE	Decreased	40P and 22HC	(85)				
		T1D	Decreased	11P and 22HC	(113)				
***Clostridiaceae***	***SFB***	RA	Enriched			K/BxN	(102)	Exacerbates	Immune dysregulation/Tfh+Autoantibodies
***Enterobacteriaceae***	***E. coli***	IBD	Enriched	447P and 221HC	(35)	DSS	(33)	Exacerbates	Immune dysregulation/IL-17
		IBD	Enriched	21P and 7HC	(34)	DSS	(33)	Exacerbates	Immune dysregulation/IL-17
		IBD	Enriched	59P	(33)	Salmonella infection	(56)	Improves	Immune dysregulation/IL-10
***Enterococcaceae***	***E. gallinarum***	SLE	Enriched	3P and 5HC	(90)	(NZW × BXSB)F1	(90)	Exacerbates	Immune dysregulation/AhR (AhR ligands)
***Lachnospiraceae***	**Not identified**	IBD	Decreased	129P and 61HC	(16)				
		T1D	Decreased	11P and 22HC	(113)				
	***R. gnavus***	IBD	Enriched	20P and12 HC	(37)				
		SLE	Enriched	61P and 17HC	(6)				
		T1D	Enriched	415P and 267HC	(9)				
***Lactobacillaceae***	***Lactobacillus***	SLE	Enriched	20P and 19HC	(84)	Sle1, 2 and 3	(91)	Exacerbates	Immune dysregulation/AhR (Tryptophan-derivatives)
	***L. reuteri***	SLE	Enriched	12P and 22HC	(86)	TLR7.1 Tg	(86)	Exacerbates	Immune dysregulation/Type I IFN
		MS	Enriched			EAE	(109)	Improves	Immune dysregulation/AhR (Indole-related)
	***L. salivarius***	RA	Enriched	77P and 80HC	(95)				
	***L. bulgaricus***	IBD	Enriched			DSS	(71)	Improves	Immune dysregulation/AhR (AhR ligands)
	***L. murinus***	MS	Enriched			EAE	(110)	Improves	Immune dysregulation/AhR (Indole-related)
***Methanobacteriaceae***	***M. smithii***	MS	Enriched	60 P and 43 HC	(8)				
***Porphyromonadaceae***	***P. gingivalis***	RA	Enriched	65 P and 18 HC	(94)				
***Prevotellaceae***	***P. copri***	RA	Enriched	83 P and 50 HC	(96)	SKG	(97)	Exacerbates	Immune dysregulation/Th17
***Ruminococcaceae***	***F. prausnitzii***	IBD	Decreased	127 P and 87 HC	(65)				
		IBD	Decreased	26 P	(32)				

P, patient; HC, healthy control.

## Author Contributions

W-JW and DZ-R performed literature searches and with GD wrote the manuscript. All authors contributed to the article and approved the submitted version.

## Funding

This work was supported by NIH AI125264 and Rainin Foundation.

## Conflict of Interest

The authors declare that the research was conducted in the absence of any commercial or financial relationships that could be construed as a potential conflict of interest.
